# Physical and electrical properties of graphene grown under different hydrogen flow in low pressure chemical vapor deposition

**DOI:** 10.1186/1556-276X-9-546

**Published:** 2014-10-02

**Authors:** Sajjad Hussain, Muhmmad Waqas Iqbal, Jaehyun Park, Muneer Ahmad, Jai Singh, Jonghwa Eom, Jongwan Jung

**Affiliations:** 1 Nanotechnology and Advanced Materials Engineering and Graphene Research Institute, Sejong University, Seoul 143-747, South Korea; 2 Department of Physics and Graphene Research Institute, Sejong University, Seoul 143-747, South Korea

**Keywords:** Graphene synthesis, H_2_ flow, CVD, Raman, Mobility

## Abstract

Hydrogen flow during low pressure chemical vapor deposition had significant effect not only on the physical properties but also on the electrical properties of graphene. Nucleation and grain growth of graphene increased at higher hydrogen flows. And, more oxygen-related functional groups like amorphous and oxidized carbon that probably contributed to defects or contamination of graphene remained on the graphene surface at low H_2_ flow conditions. It is believed that at low hydrogen flow, those remained oxygen or other oxidizing impurities make the graphene films p-doped and result in decreasing the carrier mobility.

## Background

Since the first report of the special characteristics of graphene, a planar sheet of sp^2^ hybridized carbon atoms arranged in a honeycomb lattice with hexagonal rings, the research areas of graphene have evolved tremendously from the field of fundamental physics [1,2] to the application field in low-cost flexible transparent electronics [3], photovoltaics [4], or microelectronics devices [5-9]. However, to obtain large-area and high-crystalline graphene films, low-cost elaboration method is still a significant challenge. Micromechanical exfoliation of highly ordered pyrolytic graphite (HOPG) [10,11], although yielding a good quality graphene, is not amenable to large scale production. For the nanotechnology and microelectronics industry, high quality films with low defect density, large-scale area, and high uniformity are required. The presence of grain boundaries, disorder, point defects, wrinkles, folds, tears and cracks, and so forth can scatter the charge carriers and have detrimental effects on the electronic, thermal, and mechanical properties of grapheme [12-14]. One of the most practical methods to produce graphene is chemical vapor deposition (CVD) [15-18]. Especially, the most uniform single layer graphene can be produced via CVD over a copper substrate [15]. To obtain uniform graphene film using CVD, several factors influence its growth such as solubility of carbon in the metal-substrate, crystal structure of the metal, metals lattice plane, and other thermodynamic parameters like temperature, cooling rate, pressure of the system, type of catalyst, and the amount of flow of gases in the system. Hydrogen (H_2_) flow rate is another important parameter in CVD kinetics, and it can contribute to the improvement of graphene layer uniformity even on polycrystalline substrates. Hydrogen plays a dual role during growth on copper (Cu) substrate [19]. Without the presence of H_2_, methane (CH_4_) used as a carbon precursor should chemisorb on the Cu surface to form active carbon species, that is, (CH_x_)s (*x* = 0 ~ 3), which subsequently react to form graphene. However, such dehydrogenation reactions are not thermodynamically favorable even on Cu surface [20]. Molecular H_2_ more readily dissociates on Cu and produces active H atoms [21]. These H atoms can promote activation of physisorbed CH_4_, and lead to production of surface bound (CH_3_)s, (CH_2_)s, or (CH)s radicals. It also controls the grains' shape and dimension by etching away the weak carbon-carbon bonds [19]. So, changing the flow of H_2_ gas during growth steps affects the morphology of graphene film such as the sizes, shapes, and dimensions of graphene grain boundaries (e.g., edge, pits, zigzag) [19,22].

In this article, we observed that the H_2_ flow during the low pressure CVD also had significant effect on the electrical properties of graphene. We found that in our process conditions, H_2_ seemed to act more like as a nucleation/growth promoter and a defect suppressor (oxygen-related functional groups) of CVD-grown graphene film in our process conditions.

## Methods

Graphene samples of various sizes (ranging from 1 × 1 to 3 × 3 cm) were grown on Cu foils (50 μm thick, 99.5% metal basis) purchased from Sigma-Aldrich (St. Louis, MO, USA). The Cu foil substrate was ultrasonically degreased by being immersed in acetone, methanol, isopropyl alcohol (IPA) solution, and deionized (DI) water and then dried and baked for 5 min. After the foil was placed in a quartz chamber, the chamber was evacuated and heated up to 950°C very rapidly using halogen lamps. Annealing was performed under H_2_ and argon (Ar) atmosphere at 950°C for 15 min. The Ar flow was set at 100 sccm during the annealing step for all the samples. After that, CH_4_ gas was forced to flow at 100 sccm during the growth step, and the H_2_ flow was systematically (10 to 500 sccm) varied for different samples. Then, the chamber was cooled down to room temperature without H_2_ and CH_4_ flows. Table 1 shows the pressure of chamber during the growth. The graphene film was released by protecting the graphene sheet with polymethylmethacrylate (PMMA) solution (20 mg/mL) spin-coated on graphene/copper foil at 4,000 rpm for 30 s and dried in air. Transparent ammonium persulfate (0.1 M, Sigma-Aldrich) solution was used to etch copper. The PMMA/graphene film was transferred onto a SiO_2_/Si substrate and put into acetone for 10 h to dissolve PMMA. All the samples were thermally annealed after transferring to the SiO_2_/Si substrate under nitrogen gas protection for further characterization. The as-grown and transferred films were characterized by field emission scanning electron microscopy (FESEM; HITACHI S-4700, Hitachi Ltd., Chiyoda-ku, Japan) to check the proper growth of the graphene film. Raman spectra were recorded using a Renishaw Raman microscope (Renishaw plc, Wotton-under-Edge, UK) with an Ar laser wavelength of 514 nm, and X-ray photoelectron spectroscopy (XPS; PHI Model 5400 AXIS Ultra Kratos XPS, Kratos Analytical, Chestnut Ridge, NY, USA) was used to confirm the quality of the film. The atomic force microscopy (AFM) was done to analyze the morphology of grown films, grain boundaries, and thickness using the commercial AFM n-Tracer (NanoFocus Inc., Oberhausen, Germany). Electrical contacts were made by thermally evaporating Au/Cr with thickness of 30/5 nm for transport measurements.

**Table 1:** The pressure of chamber during the growth

**H**_**2 **_**flow rate (sccm)**	**Total pressure/H**_**2 **_**partial pressure (mTorr)**
10	353/16.8
100	792/364
200	980/264
300	998/598.8
400	2,470/1,646.7
500	2,780/1,985.7

Ar/H_2_/CH_4_ = 100/10 ~ 500/100 sccm.

## Results and discussion

**Figure 1:**
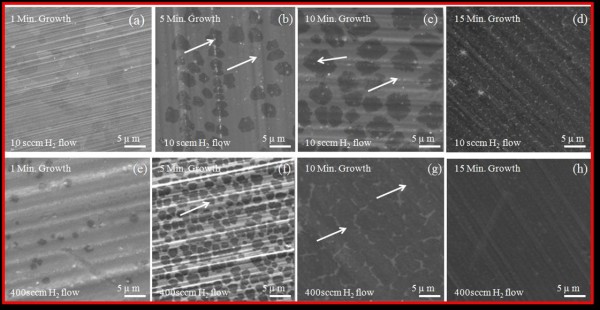
**SEM micrographs of obtained grown graphene samples.** At 950°C for 1, 5, 10, and 15 min at constant 10 and 400 sccm H_2_ flow **(a-h)**.

**Figure 2:**
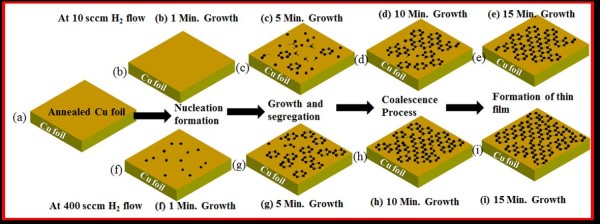
**Illustration of growth process of CVD graphene on Cu.** At 950°C for 1, 5, 10, 15 min at constant 10 and 400 sccm H_2_ flow **(a-i)**. Illustrative growth schematics: (i) incubation, (ii) nucleation formation, (iii) growth and segregation, (iv) coalescence process, and (v) formation of films.

A large number of graphene samples were prepared, corresponding to the H_2_ flow of 10, 100, 200, 300, 400, and 500 sccm at four different growth durations of 1, 5, 10, and 15 min. Figure 1 shows the SEM micrographs of the as-grown graphene samples obtained at 950°C at two different H_2_ flow rates of 10 (low H_2_ flow) and 400 sccm (high H_2_ flow). The corresponding growth schematics during each elapse time are shown in Figure 2.

As shown in Figure 1a, at low H_2_ flow rate (10 sccm), no nucleation occurs for smaller growth durations, e.g., *t* < 5 min. The spontaneous nucleation and subsequent growth of graphene occurs only when a critical level of supersaturation is reached. At lower H_2_ flow rates and hence at low partial pressure, this critical level of supersaturation is achieved only at longer growth durations. As the H_2_ flow rate increases, this threshold of supersaturation is achieved at relatively shorter growth durations. Further, our results also suggest that as the H_2_ flow increases the density of nuclei increases (Figure 1b,f). At the growth and segregation step (Figure 1c,g), the graphene growth rate is visibly influenced by the H_2_ flow rate, which is clearly visible in the SEM pictures (Figure 1b,f). Whereas when the H_2_ flow rate is very low (10 sccm), the nucleation and growth are reduced significantly, as compared in Figure 1b,c,f,g. The grown graphene films consist of irregular-shaped grains of different sizes at our CVD conditions. It has been reported that at low H_2_ partial pressure, the graphene domain feature irregular shapes [19]. The domain and dislocation formation during graphene growth influences the electrical properties.

**Figure 3:**
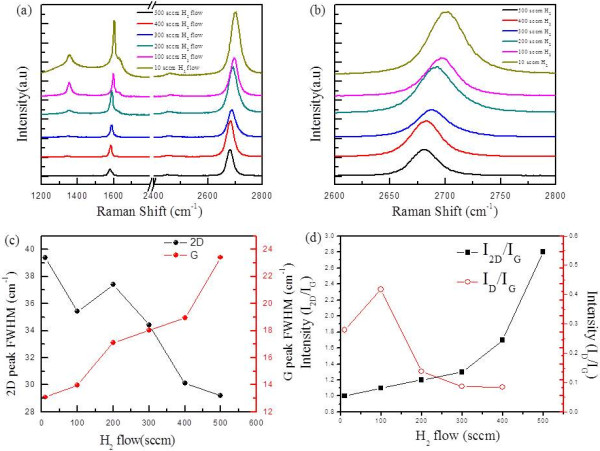
**Raman analysis of graphene films at different H**_**2 **_**flow. (a)** Raman spectra. **(b)** Zoom in approximately 2,700 cm^-1^ peak (2D) region. **(c)** The FWHM of G and 2D peaks. **(d)***I*_2D_/*I*_G_ and *I*_D_/*I*_G_.

To check the crystallinity of graphene, we used Raman spectroscopy on all the prepared samples corresponding to H_2_ flow (10 to 500 sccm) at a growth time of 15 min. As can be observed from Figure 3a, we found that as the H_2_ flow increases from 10 to 500 sccm, the intensity of defect peak in the Raman spectra decreases and is finally unnoticeable for 500 sccm of H_2_, indicating that the quality of grown graphene increases with increasing H_2_ flow. The D peak is located at approximately 1,350 cm^−1^, attributed to the breathing mode of sp^2^ atoms, and originated by the existence of some defects such as edges, grain boundaries, functional groups, or structural disorders [23]. As for the sample grown at 10 sccm, the peak positions of G, 2D, and D appear around 1,608, 2,698, and 1,357 cm^−1^, respectively. The G peak becomes sharp, and the D peak shows high intensity. While for the sample grown at high H_2_ flow (500 sccm), the peak positions of G and 2D are red shifted to around 1,585 and 2,683 cm^−1^, respectively, as can be observed in Figure 3b. The full width half maximum (FWHM) of 2D peak is also decreased with increasing H_2_ flow, as can been seen in Figure 3c, which confirms the crystalline quality of graphene film improved at a higher flow of H_2_. The FWHM of 2D peak is 28 to 39 cm^−1^ for all the samples, as can be seen in Figure 3c. The *I*_D_/*I*_G_ ratio decreases from 0.42 to 0.08, whereas the *I*_2D_/*I*_G_ ratio increases from 1.0 to 1.7 with increase of H_2_ flow from 10 to 400 sccm as can be presented in Figure 3d. At a temperature of 900°C, the diffusion coefficient of H_2_ in Cu is 2 × 10^−4^ cm^2^s^−1^. H_2_ or atomic H can easily diffuse into the catalyst and compete with CH_4_ for chemisorption; H_2_ can also contribute to the fast removal of residual oxygen and contaminants from the surface [19]. These H_2_ effects seem to be beneficial in improving the qualities of graphene in our process conditions.

**Figure 4:**
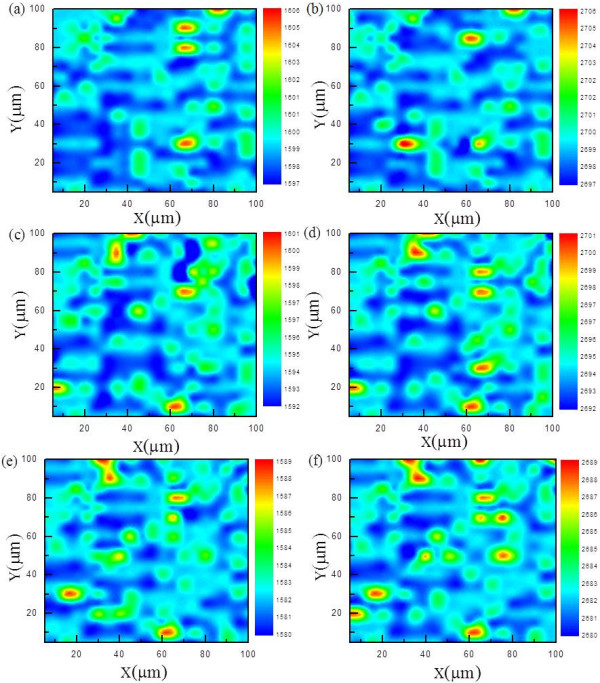
**Raman mapping of graphene film at different H**_**2 **_**flow. (a-b)** 10, **(c-d)** 200, and **(e-f)** 500 sccm G and 2D peaks.

**Figure 5:**
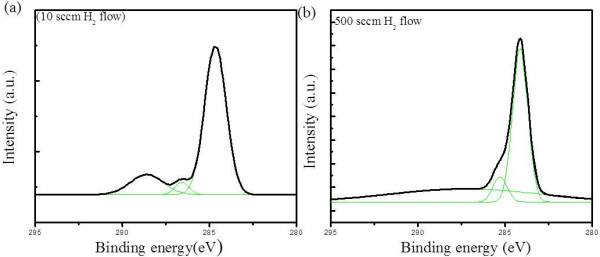
**XPS spectra of CVD-grown graphene film at H**_**2 **_**flow of (a) 10 and (b) 500 sccm.**

To further examine the thickness uniformity of graphene film at the micrometer scale, 2D and G bands were taken over an area of 100 × 100 μm at 300 different locations (Figure 4). The 2D peak positions appeared between 2,697 to 2,706, 2,692 to 2,701, and 2,680 to 2,689 cm^−1^, while G peaks appeared between 1,597 to 1,606, 1,592 to 1,601, and 1,580 to 1,589 cm^−1^ for samples at 10, 200, and 500 sccm of H_2_ flow, respectively (Figure 4a,b,c,d,e,f). It is clear that both G and 2D peak positions are shifted as H_2_ flow rate changes. The G and 2D peak shift is closely related to the graphene doping. The peak positions become both blue shifted as graphene is p-doped and red shifted when n-doped [24-28]. Since the G and 2D peak positions of the pristine CVD-graphene are reported to appear around 1,590 and 2,688 cm^−1^, respectively [23]. The sample grown at high H_2_ flow seems to be close to pristine graphene, and those grown at lower H_2_ flow seems to be p-doped, somehow based on the Raman spectra. Figure 5 shows the XPS of graphene films grown under different H_2_ flows of 10 and 500 sccm for 15 min.

C1s XPS spectra display an asymmetric shape typical of graphitic sp^2^ carbon with binding energy of 284.1 to 284.6 eV. The remaining spectra can be adequately fitted by two different Gaussian contributions: C-C sp^3^ at hydroxyl C-OH (285.9 to 286.2 eV) and carbonyl C = O and carboxylic COOH (288.3 to 288.7 eV) groups [29,30]. The sp^2^ hybridization is attributed to the carbon lattice, while sp^3^ is a result of oxygen-related functional groups like amorphous and oxidized carbon that probably contributed to defects or contamination [31]. The sample grown at high H_2_ flow (500 sccm) shows higher sp^2^/sp^3^ peak ratio than that grown at 10 sccm in Figure 5 (b). A sharp graphitic peak sp^2^ is observed at 284.6 eV with FWHM of 0.69 eV, while sp^3^ peaks at hydroxyl C-OH and carboxylic COOH are at 285.3 and 288.1 eV, respectively. As for the sample grown at low H_2_ flow (10 sccm), the sp^2^/sp^3^ ratio decreases significantly, and sp^2^ peak is broadened, which reveals that graphene is oxidized and amorphized at the low H_2_ atmosphere. It is also thought that residual oxygen or oxidizing impurities [32], which are originally remained in the CVD chamber and gas feedstock, make the graphene films p-doped at low H_2_ atmosphere.

**Figure 6:**
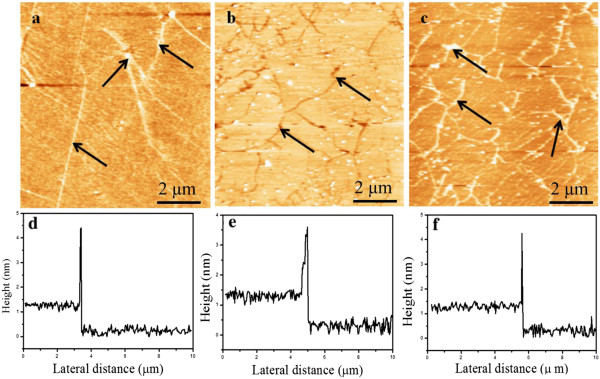
**AFM topographical maps and height profiles of samples grown at H**_**2 **_**flow of (a, d) 10, (b, e) 200, and (c, f) 400 sccm.**

AFM was performed at ambient conditions on the transferred graphene samples on to Si/SiO_2_. Panels in Figure 6a,b,c show the topography images of the samples grown at 10, 200, and 400 sccm of H_2_ each for a growth time of 15 min. As can be observed from the topography maps (Figure 6a,b,c) for all the films, the graphene domains are fully grown to merge with each other at the domain boundaries indicated by arrows (black), which confirms the graphene covering all of the substrate without gaps. In Figure 6d,e,f, the line profiles correspond to the topography maps taken at the graphene substrate interface (not shown here). We can see the thickness of approximately 1 nm on average, which confirms nearly single layer graphene. Some high peaks are visible in the line profiles which correspond to the white particles and may be some dirt particles (as the measurements were done at ambient conditions).

**Figure 7:**
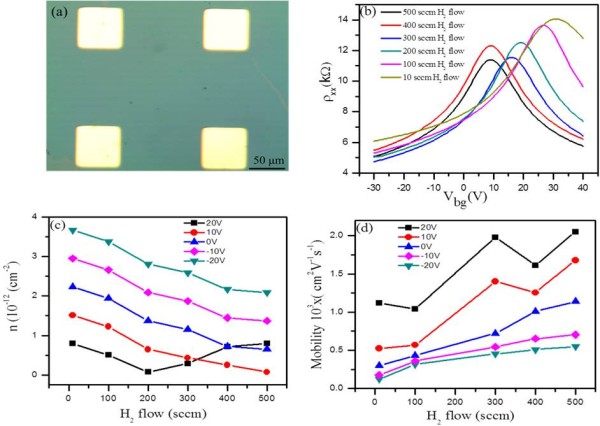
**Electrical properties of the graphene samples. (a)** Optical image of graphene based device. **(b)** Resistivity as a function of gate voltage (*V*_g_) for the graphene device. **(c-d)** Carrier concentration and mobility at different gate voltages (Δ*V*_g_ = −20, −10, 0, +10, +20 V).

To further confirm the quality of the graphene films, charge transport measurements were performed. Figure 7b shows the resistivity (ρ) of six representative samples as a function of gate voltage (*V*_g_) under different H_2_ flows. In principle, the charge neutrality point (CNP) should be at *V*_g_ = 0 if the graphene is undoped [33,34]. The charge carrier density as a function of gate voltage can be obtained from the relation *ne* = *C*_g_(*V*_g_ − *V*_Dirac_), where *C*_g_ is the gate capacitance for a SiO/Si substrate [35]. In order to quantitatively analyze the difference between samples, we employed the semiclassical Drude [34] model to estimate the mobility of samples *μ* = (*enρ*)^− 1^.

In the experiments, more than six devices were made. There is a trend in which the mobility of the graphene increases and the CNP shifts toward zero with increasing H_2_ flow rate. For the sample grown under 10 sccm of H_2_ flow, the CNP was at +31 V, but for the sample under 500 sccm H_2_ flow, the CNP was shifted to +10 V. Our results show that at low H_2_ flow all the film exhibited p-typed behavior [36]. With the increasing the H_2_ flow, the synthesized graphene becomes closer to a pristine graphene, and its mobility increases. Figure 7c shows the charge carrier densities significantly change with different H_2_ flow. The changes in charge carrier density of graphene layers are related with changes in Fermi level of graphene layers. Thus, different H_2_ flow significantly modulates the Fermi level of graphene layers. The defects of graphene, which can be originated from the inevitable occurrence of residual oxidizing impurities in the chamber's atmosphere and gas feedstock, are suppressed under high H_2_ flow rate.

**Figure 8:**
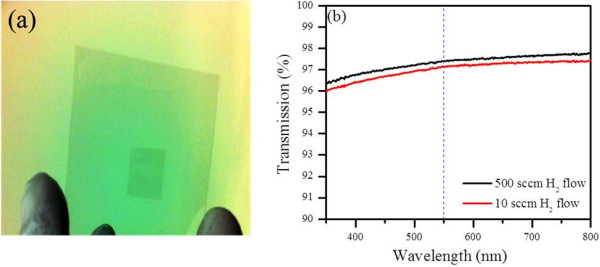
**Transmittance data of graphene films. (a)** Photograph of graphene films on glass **(b)**. Transmittance spectra.

The optical transmittance spectra as depicted in Figure 8b show that the transmittance values at 550 nm are almost same regardless of H_2_ flow and is in good agreement with that reported for a monolayer graphene (97.1%), indicating all monolayer graphene films as confirmed from the AFM topography [37]. Our results will hopefully contribute in growing defect-free crystalline graphene which will be directly applicable in transparent nanoelectronics.

## Conclusions

In conclusion, we observed that hydrogen flow during the low pressure chemical vapor deposition had significant effect not only on the physical properties but also on the electrical properties of graphene. Nucleation and grain growth of graphene increased at higher hydrogen flows. And, more oxygen-related functional groups like amorphous and oxidized carbon that probably contributed to defects or contamination of graphene remained on the graphene surface at low H_2_ flow conditions. It is believed that at low hydrogen flow, those remained oxygen or other oxidizing impurities make the graphene films p-doped and result in decreasing the carrier mobility. In our process conditions, H_2_ seemed to act more as a nucleation/growth promoter and a defect suppressor (oxygen-related functional groups) of CVD-grown graphene film.

## Competing interests

The authors declare that they have no competing interests.

## Authors’ contributions

SH and JP performed the experiments. MI, MA, JS, and JE analyzed the data. SH and JJ wrote the paper. All authors read and approved the final manuscript.
